# Supplementary figures and images for: Genome-wide identification and response stress expression analysis of the BES1 family in rubber tree (Hevea brasiliensis Muell. Arg.)
Source: PeerJ. 2022 May 13;10:e13189. doi: 10.7717/peerj.13189 (PMC9109691; doi:10.7717/peerj.13189)

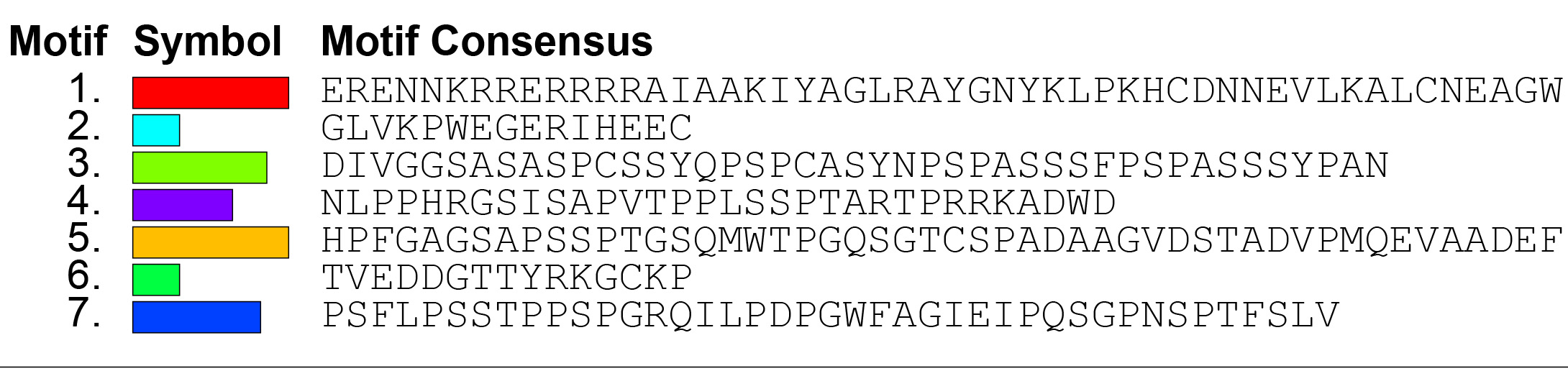

Supplement: Supplemental Information 1 — Colored boxed represent different motifs. Letters in right represent conserved amino acids of motifs. [file peerj-10-13189-s001.jpg]
