# Supplementary material for: Genome-wide identification and response stress expression analysis of the BES1 family in rubber tree (Hevea brasiliensis Muell. Arg.)
Source: PeerJ. 2022 May 13;10:e13189. doi: 10.7717/peerj.13189 (PMC9109691; doi:10.7717/peerj.13189)
Supplement: Supplemental Information 3 [file peerj-10-13189-s003.docx]

Table S1 List of HbBES1 family primers used for quantitative polymerase chain reaction gene expression

| Primer name | Forward primer sequence (5'-3') | Reverse primer sequence (5'-3') |
| --- | --- | --- |
| *HbBES1-1* | CTCCCTAAGCACTGCGACAA | CCATGCGTTCCACAGGTTTG |
| *HbBES1-2* | CACTCTGCAATGAGGCTGGT | AGCACAAGGACTTGGTTGGT |
| *HbBES1-3* | CTCCAGCCACCATACCTGAA | AGGTTGGAGAGGTTGGCATT |
| *HbBES1-4* | GCAAGTCCGTGCTCATCCTA | GCAGTTGGCGAGCTTAATGG |
| *HbBES1-5* | GGCTTGAGTTGGCAGGTCTT | AAGGTGATGGCTGCTGGTAG |
| *HbBES1-6* | TGAGAGGAGAGCTTGCTGC | GGTAGGAAGGTACAGGACTGG |
| *HbBES1-7* | GACGAGGCTACCAACATGGA | AATATCCACGCGCTCCACAG |
| *HbBES1-8* | GAGGCTGGTTGGATCGTTGA | TGCGGAGGAATGGAAGAAGG |
| *HbBES1-9* | AAGTGTGAGCTGGCTGATCC | AGCGTGTGCCTCCACTATTC |
| *HbActin* | GATGTGGATATCAGGAAGGA | CATACTGCTTGGAGCAAGA |
