# Supplementary material for: Genome-wide identification and response stress expression analysis of the BES1 family in rubber tree (Hevea brasiliensis Muell. Arg.)
Source: PeerJ. 2022 May 13;10:e13189. doi: 10.7717/peerj.13189 (PMC9109691; doi:10.7717/peerj.13189)
Supplement: Supplemental Information 4 [file peerj-10-13189-s004.docx]

Table S2 Cis-elements and corresponding functions of HbBES1 promoters

| *Cis*-elements | Function |
| --- | --- |
| GARE-motif | gibberellin-responsive element |
| ABRE | cis-acting element involved in the abscisic acid responsiveness |
| AuxRE | cis-acting regulatory element involved in auxin responsiveness |
| CAT-box | cis-acting regulatory element related to meristem expression |
| G-box | cis-acting regulatory element involved in light responsiveness |
| MSA-like | cis-acting element involved in cell cycle regulation |
| MYB | binging with MYB |
| MYC | binding with MYC |
| P-box | gibberellin-responsive element |
| Sp1 | gibberellin-responsive element |
| TGACG-motif | cis-acting regulatory element involved in the MeJA-responsiveness |
| TGA-motif | auxin-responsive element |
| ARE | cis-acting regulatory element essential for the anaerobic induction |
| GT1-motif | light responsive element |
| MBS | MYB binding site involved in drought-inducibility |
| MRE | MYB binding site involved in light responsiveness |
| TCA-element | cis-acting element involved in salicylic acid responsiveness |
| WUN-motif | wound-responsive element |
| TATC-box | cis-acting element involved in gibberellin-responsiveness |
| CCAAT-box | MYBHv1 binding site |
| 3-AF1 binding site | light responsive element |
| AT-rich element | binding site of AT-rich DNA binding protein (ATBP-1) |
